# 5Z-7-Oxozeanol Inhibits the Effects of TGFβ1 on Human Gingival Fibroblasts

**DOI:** 10.1371/journal.pone.0123689

**Published:** 2015-04-30

**Authors:** Hanna Kuk, James Hutchenreuther, Hannah Murphy-Marshman, David Carter, Andrew Leask

**Affiliations:** 1 Department of Physiology and Pharmacology, The University of Western Ontario, Schulich School of Medicine and Dentistry, London, Ontario, Canada, N6A 5C1; 2 Department of Dentistry, The University of Western Ontario, Schulich School of Medicine and Dentistry, London, Ontario, Canada, N6A 5C1; 3 London Regional Genomics Centre, Robarts Research Institute, London, ON, Canada, N6A 5B7; Boston University Goldman School of Dental Medicine, UNITED STATES

## Abstract

Transforming growth factor (TGF)β acts on fibroblasts to promote the production and remodeling of extracellular matrix (ECM). In adult humans, excessive action of TGFβ is associated with fibrotic disease and fibroproliferative conditions, including gingival hyperplasia. Understanding how the TGFβ1 signals in fibroblasts is therefore likely to result in valuable insights into the fundamental mechanisms underlying fibroproliferative disorders. Previously, we used the TAK1 inhibitor (5Z)-7-Oxozeaenol to show that, in dermal fibroblasts, the non-canonical TAK1 pathway mediates the ability of TGFβ1 to induce genes promoting tissue remodeling and repair. However, the extent to which TAK1 mediates fibroproliferative responses in fibroblasts in response to TGFβ1 remains unclear. Herein, we show that, in gingival fibroblasts, (5Z)-7-Oxozeaenol blocks the ability of TGFβ1 to induce expression of the pro-fibrotic mediator CCN2 (connective tissue growth factor, CTGF) and type I collagen protein. Moreover, genome-wide expression profiling revealed that, in gingival fibroblasts, (5Z)-7-Oxozeaenol reduces the ability of TGFβ1 to induce mRNA expression of essentially all TGFβ1-responsive genes (139/147), including those involved with a hyperproliferative response. Results from microarray analysis were confirmed using real time polymerase chain reaction analysis and a functional cell proliferation assay. Our results are consistent with the hypothesis that TAK1 inhibitors might be useful in treating fibroproliferative disorders, including that in the oral cavity.

## Introduction

Wound healing is a highly regulated process that occurs in all tissues and organs of the body in response to injury. Excessive deposition and remodeling of connective tissue can result in fibroproliferative conditions [1], which, in adult tissues, can be characterized by the presence of scar tissue or pathological fibrosis. Scars replace normal tissue architecture thus diminishing the function of the tissue or organ. It is estimated that 45% of deaths in the developed countries are attributed to some form of pathological fibrosis [2]. The effector cell of pathological scarring is the myofibroblast, a type of fibroblast characterized by the presence of αsmooth muscle actin (SMA)-containing stress fibers [3]. Intriguingly, fibrotic responses in the oral cavity do not involve either the deposition of scar tissue or the presence of abundant myofibroblasts, but are instead characterized by an excessive hyperproliferative response that results in gingival overgrowths, for example, in response to antiepileptic medications, calcium channel blockers and immunosuppressant drugs [4]. Thus comparing the signaling responses of dermal and gingival fibroblasts to fibrogenic stimuli is of inherent value.

TGFβ1 is a potently fibrogenic growth factor which promotes the ability of fibroblasts to proliferate, migrate, deposit and remodel newly formed extracellular matrix (ECM). TGFβ1-mediated signaling involves both canonical (Smad-dependent) and non-canonical (Smad-independent) pathways [5]. The former mediates essentially all cellular responses to TGFβ1 [5]. For example, previously we and others have shown that the canonical ALK5/Smad3 pathway mediates pro-fibrotic responses to TGFβ in a variety of fibroblasts, including the ability of TGFβ to induce expression of the profibrotic marker CCN2 in both dermal and gingival fibroblasts [6–10].

One non-canonical TGFβ pathway is mediated by TGFβ-associated kinase 1 (TAK1), a mitogen-activated kinase kinase kinase (MAP3K), which is crucial for the activation of the p38 and JNK MAPK pathways [11]. In human adult dermal and mouse embryonic fibroblasts, TAK1 pathway selectively mediates adhesive, migratory, proliferative and contractile responses to TGFβ1 [12, 13]. Genome-wide expression profiling showed that the TAK1 inhibitor (5Z)-7-Oxozeaenol blocked the induction of ~70% of the TGFβ1-responsive mRNAs in human adult dermal fibroblasts [13]. However, whether TAK1 mediates the fibroproliferative responses to TGFβ1 in gingival fibroblasts is unknown.

To address this gap in our knowledge, in this report we test whether the selective TAK1 inhibitor 5Z-7-Oxozeanol inhibits the ability of TGFβ1 to induce fibroproliferative responses in cultured gingival fibroblasts.

## Methods

### Cell Culture and Ethics Statement

Previously isolated gingival fibroblast cells generated according to an approved ethical protocol at the University of Western Ontario [6] were grown in high glucose DMEM, 10% FBS and 1% antibiotic-antimycotic (Invitrogen) at 37°C, 5% CO_2._ Cells were cultured in 96 well plates (for proliferation assays) or 6 well plates (for all other assays) until 40–60% confluence. Cells were then cultured overnight in low glucose DMEM, 0.5% FBS, and pre-treated with DMSO or 400 nM (5*Z*)-7-Oxozeaenol (Tocris; a concentration previously shown to be selective for TAK1 [13–15]) for 45 minutes prior to treatment with or without TGFβ1 (4 ng/ml, R&D systems). Cultures of passages 8 through 10 were utilized for this study.

### RNA Extraction and Real-Time RT-qPCR

Real time PCR was conducted essentially as previously described [6]. Total RNAs were extracted (Trizol, Invitrogen) 6 hours post-addition of TGFβ1 and the concentration and integrity of the extracted RNA sample was measured using a Nanodrop 2000 (Thermo) and Agilent 2100 Bioanalyzer (Agilent Technologies Inc., Palo Alto, CA) using the RNA 6000 Nano kit (Caliper Life Sciences, Mountain View, CA). The extracted RNA samples (50 ng) were reversed transcribed and amplified using TaqMan Human gene Expression assays (Applied Biosystems) in a 15-μL reaction containing qScript™ XLT One-Step RT-qPCR ToughMix (Quanta Biosciences) TaqMan Assays-on-demand Human gene specific primers (Applied Biosystems), 6-carboxyflurosceinlabeled gene specific TaqMan MGB probe (Applied Biosystems). The ViiA™ 7 Real-Time PCR System and ViiA™ 7 Software were used for the detection and analysis of the amplified signal according to manufacturer’s instructions (Applied Biosystems). Triplicate samples were run, and experiments were repeated on three independent occasions, and averages +/- SEM (N = 3) calculated. Single factor ANOVA and Tukey's Post Hoc analysis were used for statistical analysis (GraphPad Prism software).

### Western Blot Analysis

Proteins were harvested 24 hours post-addition of TGFβ1 in RIPA buffer (100 mM Tris HCl, pH 7.4; 150 mM NaCl; 1% NP40; 0.1% SDS; 5 mM EDTA, 1X PMSF, 1X protease inhibitor cocktail) and total protein concentration of the lysates was established using the BCA assay microplate procedure as per manufacturer instructions (Pierce). When phosphoproteins were to be detected, protein extraction in the presence of 1mM Sodium orthovanadate, 1mM sodium fluoride, and 2.5mM β-glycerolphosphate was performed. Proteins (50 μg) were then subjected to SDS-PAGE using a 10% acrylamide gel. Gels were then transferred onto nitrocellulose membrane (iBlot; Invitrogen). The resultant membranes were incubated for 1 hour in Blocking buffer solution (50 mM Tris, 150 mM NaCl; 0.05% Tween-20; 5% skim milk) followed by the addition of anti-CCN2 (L-20) goat polyclonal primary antibody (1:250; Santa Cruz Biotechnology) or anti-type I collagen antibody (1:4000) and subsequent overnight incubation at 4°C. Alternatively, blots were probed with anti-phospho-TAK1 antibody (1:1000: Abcam). Membranes were washed three times in TBST at room temperature for 10 min and incubated with an anti-goat secondary HRP conjugated polyclonal antibody (1:2000; Jackson Immunoresearch) for one hour at room temperature and imaged on X-ray film (Kodak) with the use of Chemiluminescent Substance (1:1 Lumino/Enhancer solution to Stable Peroxide Solution ratio; Thermo Scientific). Membranes were subsequently washed in TBST and stripped with the Restore western blot stripping buffer (Thermo) for 20 min at room temperature, blocked and re-probed with β-actin mouse monoclonal primary antibody (Sigma; 1:8000 dilution) overnight at 4°C in TBST prior to exposure with appropriate HRP conjugated secondary antibody. Densitometry measures for band intensities for TGFβ1 and TGFβ1 + (5*Z*)-7-Oxozeaenol treatment groups were obtained using ImageJ program (NIH) and standardized to the respective β-actin levels. Student’s t-test was used for statistical analysis (GraphPad Prism software).

### Indirect Immunofluorescence Analysis

Cells cultured on glass coverslips (VWR) were fixed in 4% paraformaldehyde in PBS, permeabilized with 0.2% TritonX100 (Sigma-Aldrich) in PBS and blocked with 5% Donkey serum in PBST (10 mM phosphate buffer, 2.7 mM KCl, 137 mM NaCl, 0.05% Tween 20; pH 7.4) for 30 min at room temperature followed by the addition of CCN2 (L-20) goat polyclonal antibody (1:100; Santa Cruz Biotechnology). Following one hour incubation at room temperature, the samples were washed in PBS and incubated with DyLight 594 anti-goat IgG secondary antibody in 5% Donkey serum in PBST for 45 min at room temperature in the dark (1:1000; Jackson ImmunoResearch Laboratories). The coverslips were mounted on the slides using VECTASHIELD Mounting Media (Vector Laboratories). Images were taken with a Zeiss Axio Imager.M1 microscope and Northern Eclipse software. Total fluorescence intensity was obtained for each individual image and divided by the representative cell number using DAPI stain as a guide. Relative fluorescence intensity ratio was further obtained using DMSO treatment group as a standard. Single factor ANOVA and Tukey's Post Hoc analysis were used for statistical analysis (GraphPad Prism software).

### Gene Expression Profiling

Gene expression profiling was conducted essentially as previously described [13]. All sample labeling and GeneChip processing was performed at the London Regional Genomics Centre (Robarts Research Institute, London, Ontario, Canada; http://www.lrgc.ca). RNA quality was assessed using the Agilent 2100 Bioanalyzer (Agilent) and the RNA 6000 Nano kit (Caliper Life Sciences). Single stranded complimentary DNA (sscDNA) was prepared from 200 ng of total RNA as per the Ambion WT Expression Kit for Affymetrix GeneChip Whole Transcript WT Expression Arrays (http://www.ambion.com/techlib/prot/fm_4411973.pdf, Applied Biosystems, Carlsbad, CA) and the Affymetrix GeneChip WT Terminal Labeling kit and Hybridization User Manual (http://media.affymetrix.com/support/downloads/manuals/wt_term_label_ambion_user_manual.pdf, Affymetrix, Santa Clara, CA). Total RNA was first converted to cDNA, followed by *in vitro* transcription to make cRNA. 5.5 μg of single stranded cDNA was synthesized, end labeled and hybridized, for 16 hours at 45°C, to Human Gene 1.0 ST arrays. All liquid handling steps were performed by a GeneChip Fluidics Station 450 and GeneChips were scanned with the GeneChip Scanner 3000 7G (Affymetrix, Santa Clara, CA) using Command Console v1.1. Probe level (.CEL file) data was generated using Affymetrix Command Console v1.1. Probes were summarized to gene level data in Partek Genomics Suite v6.6 (Partek, St. Louis, MO) using the RMA algorithm (Irizarry et al., 2003). Partek was used to determine gene level ANOVA p-values and fold changes. Per previous publications [13, 16], gene lists were created using a filter of 1.7 fold change and p-value of < 0.05. Gene Ontology enrichment was performed using a Fisher’s Exact test. GEO accession number is GSE65069.

### Proliferation Assay

For the cell proliferation assay, cells (500 cells/well) were seeded in 96-well plates (Greiner Bio-One) and cultured for one day in high glucose DMEM media, 10% FBS. A “no-cell control” was also used, involving media alone. Cultures were serum-starved (low glucose DMEM; 0.5% FBS) overnight, pre-treated with DMSO (vehicle control) or 400 nM (5*Z*)-7-Oxozeaenol for 45 minutes, and then treated with or without TGFβ1 (4 ng/ml). BrdU reagent (1X, Cell Signaling) was then added to all treatment groups. Each treatment group consisted of 4 separate wells. Cultures were incubated for zero, 24, 48 and 72 hours and subjected to a colorimetric BrdU proliferation assay (Cell Signalling) and absorbance values reading obtained at 450 nm was calculated (iMark microplate absorbance reader, BioRad). Values obtained from the “no-cell control” was subtracted from each individual well. The experiment was repeated two additional times for a total of three times. A representative experiment in shown. Repeated measures ANOVA and Tukey's Post Hoc analysis were used for statistical analysis (GraphPad Prism software).

## Results

### (5*Z*)-7-Oxozeaenol reduces the ability of TGFβ1 to induce CCN2 and type I collagen mRNA and protein expression in gingival fibroblasts

**Fig 1:**
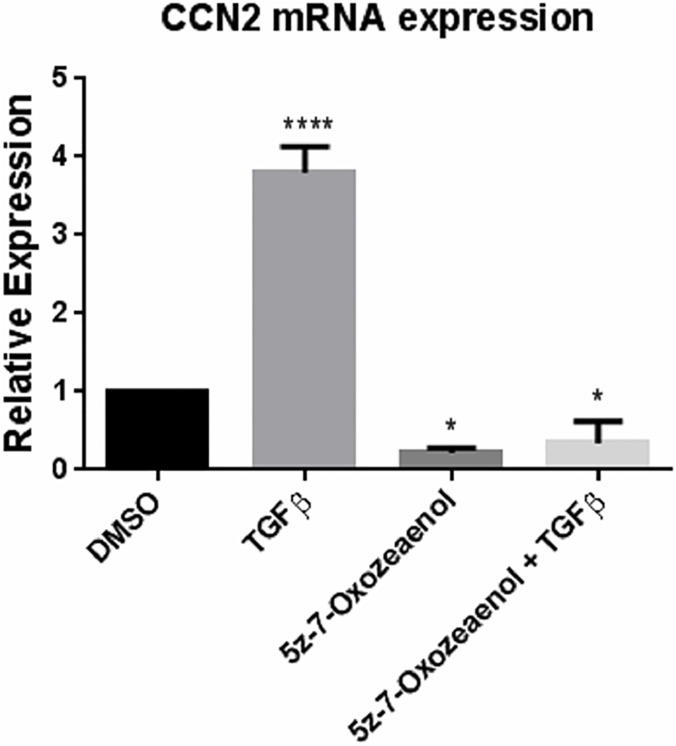
(5Z)-7-oxozeaenol reduces TGFβ1-induced CCN2 mRNA expression in human gingival fibroblasts. Human gingival fibroblasts were serum starved overnight and pre-treated with (5Z)-7-oxozeaenol (400 nM) or DMSO for 45 min followed by treatment with or without TGFβ1 (4ngml^-1^). Total RNA was harvested six hours later and subjected to TaqMan RT-qPCR analysis using the indicated probe/primer set. 18S RNA was used as the internal control. Values are expressed relative to untreated control. (N = 3; averages+/-SEM are shown; **** = p<0.0001, * = p<0.05 One-Way ANOVA).

**Fig 2:**
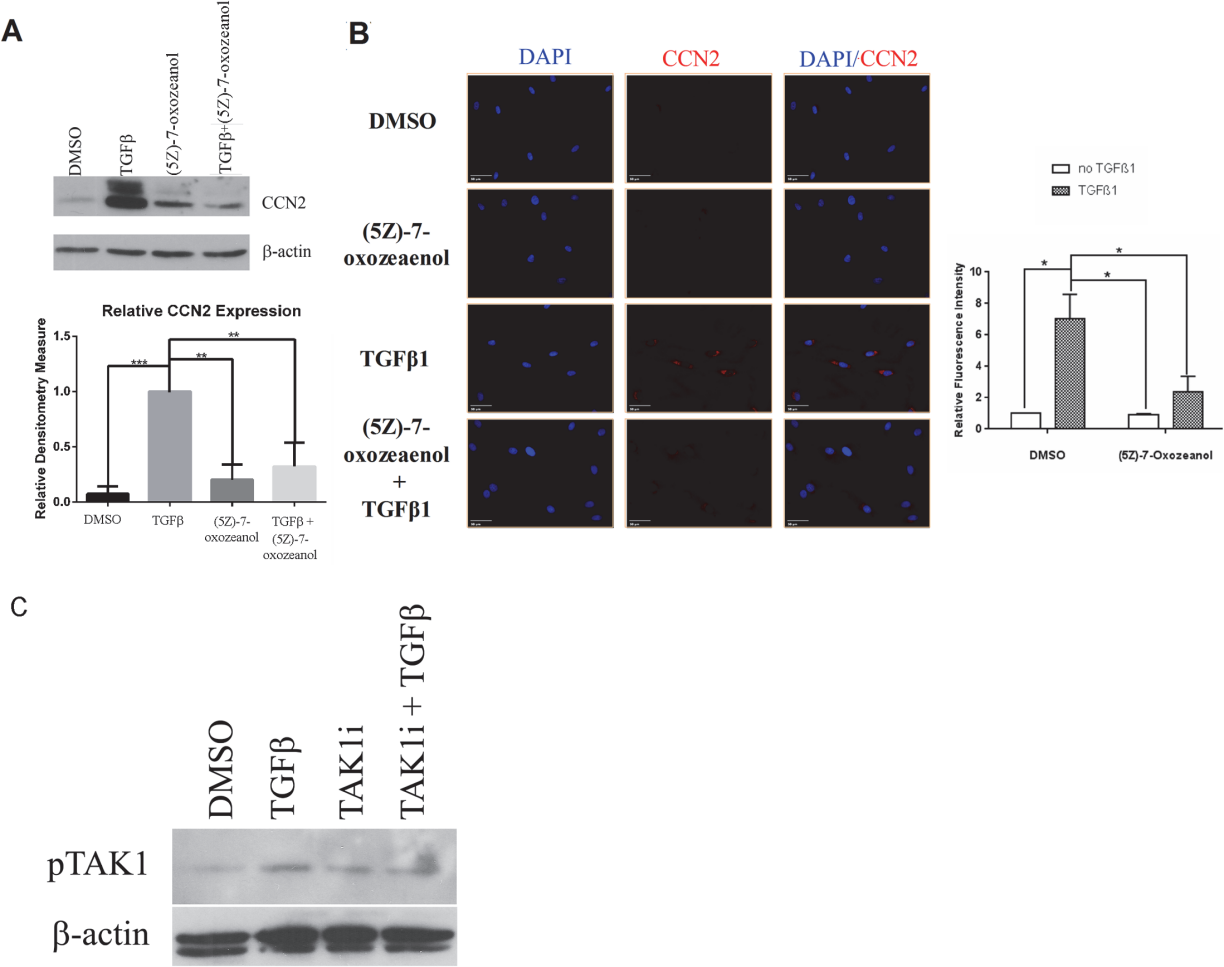
(5Z)-7-oxozeaenol reduces TGFβ1-induced CCN2 protein expression in human gingival fibroblasts. (A) Western Blot Analysis. Human gingival fibroblasts were serum starved overnight and pre-treated with (5Z)-7-oxozeaenol (400 nM) or DMSO for 45 min followed by treatment for 24 hours with or without TGFβ1 (4ng/ml). As described in methods, proteins were harvested and subjected to Western blot analysis with anti-CCN2 and anti-β-actin antibodies, as indicated. A representative blot is shown. Experiments were performed on 4 separate occasions and relative CCN2 expression in response to TGFβ1 was calculated using densitometry (N = 4, averages+/-SEM are shown; * = p<0.05, Student’s t-test. CCN2 expression in response to TGFβ was taken to represent 1). (B) Indirect immunofluorescence analysis. Human gingival fibroblasts cultured on glass coverslips as treated as in (A). Cells were fixed and stained with an anti-CCN2 antibody and DyLight 594 conjugated secondary antibody. Cells were counterstained with DAPI to detect nuclei. Representative photographs are shown. Experiments were conducted four times, and relative fluoresce intensity ratio was calculated as described in methods (N = 4, averages+/-SEM are shown. * = p<0.05, One-Way ANOVA). (C) Western Blot Analysis. Human gingival fibroblasts were serum starved overnight and pre-treated with (5Z)-7-oxozeaenol (400 nM) or DMSO for 45 min followed by treatment for 24 hours with or without TGFβ1 (4ng/ml). As described in methods, proteins were harvested and subjected to Western blot analysis with anti-phospho-TAK1 and anti-beta actin antibodies, as indicated.

**Fig 3:**
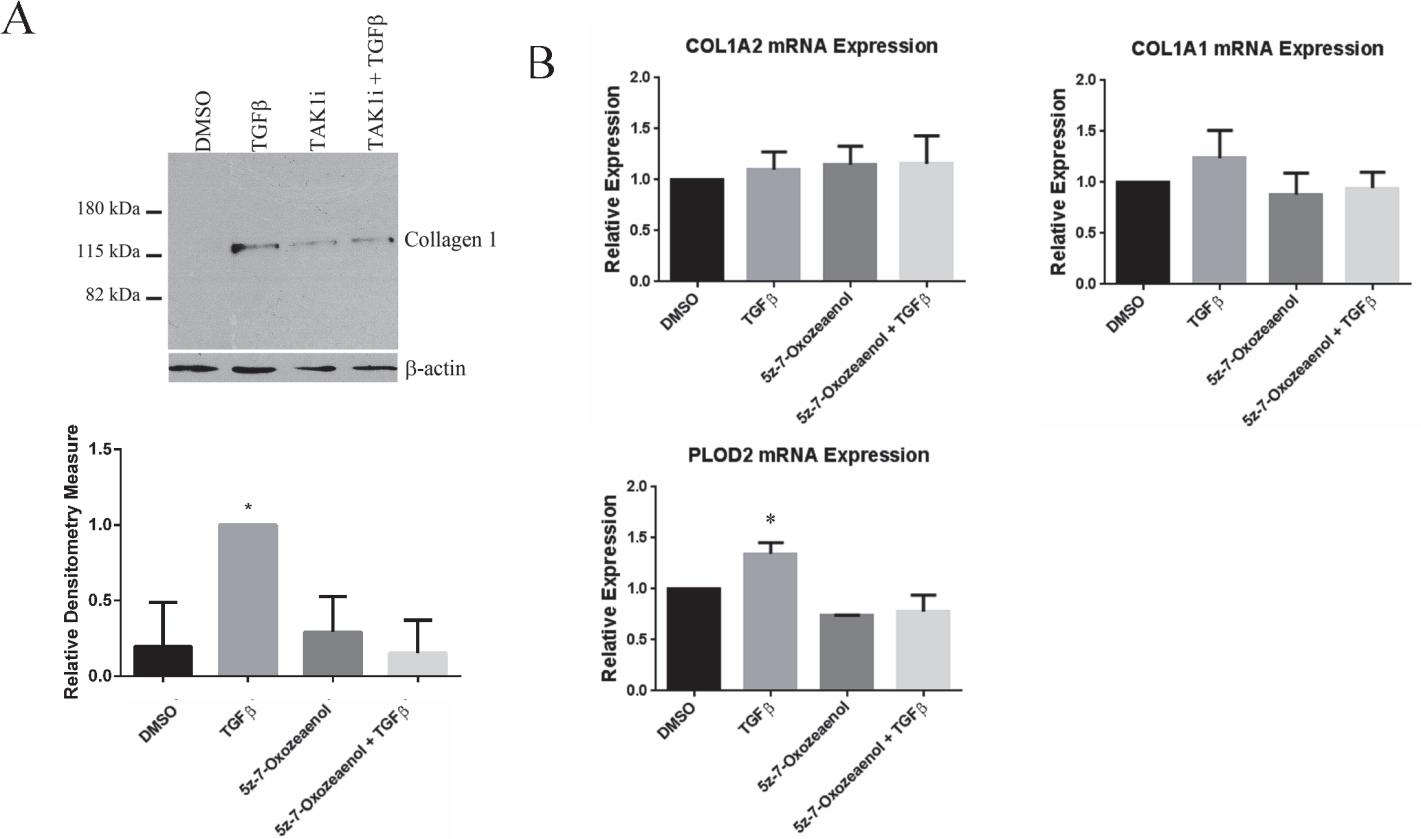
(5Z)-7-oxozeaenol reduces TGFβ1-induced collagen expression. A) Western Blot Analysis. Human gingival fibroblasts were serum starved overnight and pre-treated with (5Z)-7-oxozeaenol (400 nM) or DMSO for 45 min followed by treatment for 24 hours with or without TGFβ1 (4ng/ml). As described in methods, proteins were harvested and subjected to Western blot analysis with anti-collagen type I and anti-β-actin antibodies, as indicated. A representative blot is shown. Experiments were performed on 3 separate occasions. (N = 4, averages+/-SEM are shown; * = p<0.05, Student’s t-test. CCN2 expression in response to TGFβ was taken to represent 1). (B) mRNA analysis Human gingival fibroblasts were serum starved overnight and pre-treated with (5Z)-7-oxozeaenol (400 nM) or DMSO for 45 min followed by treatment with or without TGFβ1 (4ngml^-1^ (90 pM)). Total RNA was harvested six hours later and subjected to TaqMan RT-qPCR analysis using the indicated probe/primer set. 18S RNA was used as the internal control. (N = 3; averages+/-SEM are shown; * = p<0.05, One-Way ANOVA).

To begin to determine whether the TAK1-mediated non-canonical TGFβ signaling pathway operates in gingival fibroblasts, we first assessed whether the ability of TGFβ to induce CCN2 in gingival fibroblasts was blocked by TAK1 inhibition. We performed this analysis as an initial screening tool as CCN2, a member of the CCN family of matricellular proteins, serves as a marker and a mediator of fibrogenic responses and is highly induced by TGFβ1 [9, 17–19]. To assess whether (5*Z*)-7-Oxozeaenol, a TAK1 selective inhibitor, reduced TGFβ1-induced CCN2 expression in gingival fibroblasts, we initially used real time PCR analysis to detect CCN2 mRNA. TGFβ1 (6 hours, 4 ng/ml) significantly up-regulated CCN2 mRNA levels in gingival fibroblasts, (Fig 1). Pre-treatment of gingival fibroblasts with (5*Z*)-7-Oxozeaenol 45 minutes prior to addition of TGFβ1 significantly reduced the ability of TGFβ1 to induce CCN2 mRNA expression (Fig 1). Results were verified using Western blot analysis to detect CCN2 protein (24 h treatment with 4 ng/ml TGFβ) (Fig 2A). Similarly, indirect immunofluorescence analysis of cells using an anti-CCN2 antibody revealed that TGFβ1 (24 h treatment with 4 ng/ml TGFβ) led to an accumulation of intracellular CCN2 that was reduced by (5*Z*)-7-Oxozeaenol (Fig 2B). Similarly, TGFβ-induced type I collagen protein in gingival fibroblasts was sensitive to (5*Z*)-7-Oxozeaenol (Fig 3A). Conversely, TGFβ1 was unable to appreciably induce COL1A1 and COL1A2 mRNAs (Fig 3B), consistent with prior data that gingival fibroblasts are relatively insensitive to TGFβ1 [16] and that TGFβ1 does not potently induce COL1A1 and COL1A2 mRNAs in fibroblasts and that posttranscriptional regulation appears to be primarily responsible for TGFβ1-induced collagen type I protein expression in fibroblasts [13, 16, 20]. Consistent with this notion, TGFβ1 elevated mRNA expression of the collagen modifying enzyme procollagen-lysine, 2-oxoglutarate 5-dioxygenase (PLOD)2, which promotes collagen stability, in a (5*Z*)-7-Oxozeaenol-sensitive fashion (Fig 3B). These results suggest that the TAK1 pathway not only operates in gingival fibroblasts but is also required for TGFβ1-induced CCN2 and collagen type I expression. These results are consistent with the notion that the TAK1 pathway may mediate fibroproliferative responses to TGFββ in gingival fibroblasts.

### (5*Z*)-7-Oxozeaenol reduces the induction of essentially all TGFβ1-responsive mRNAs in gingival fibroblasts

**Fig 4:**
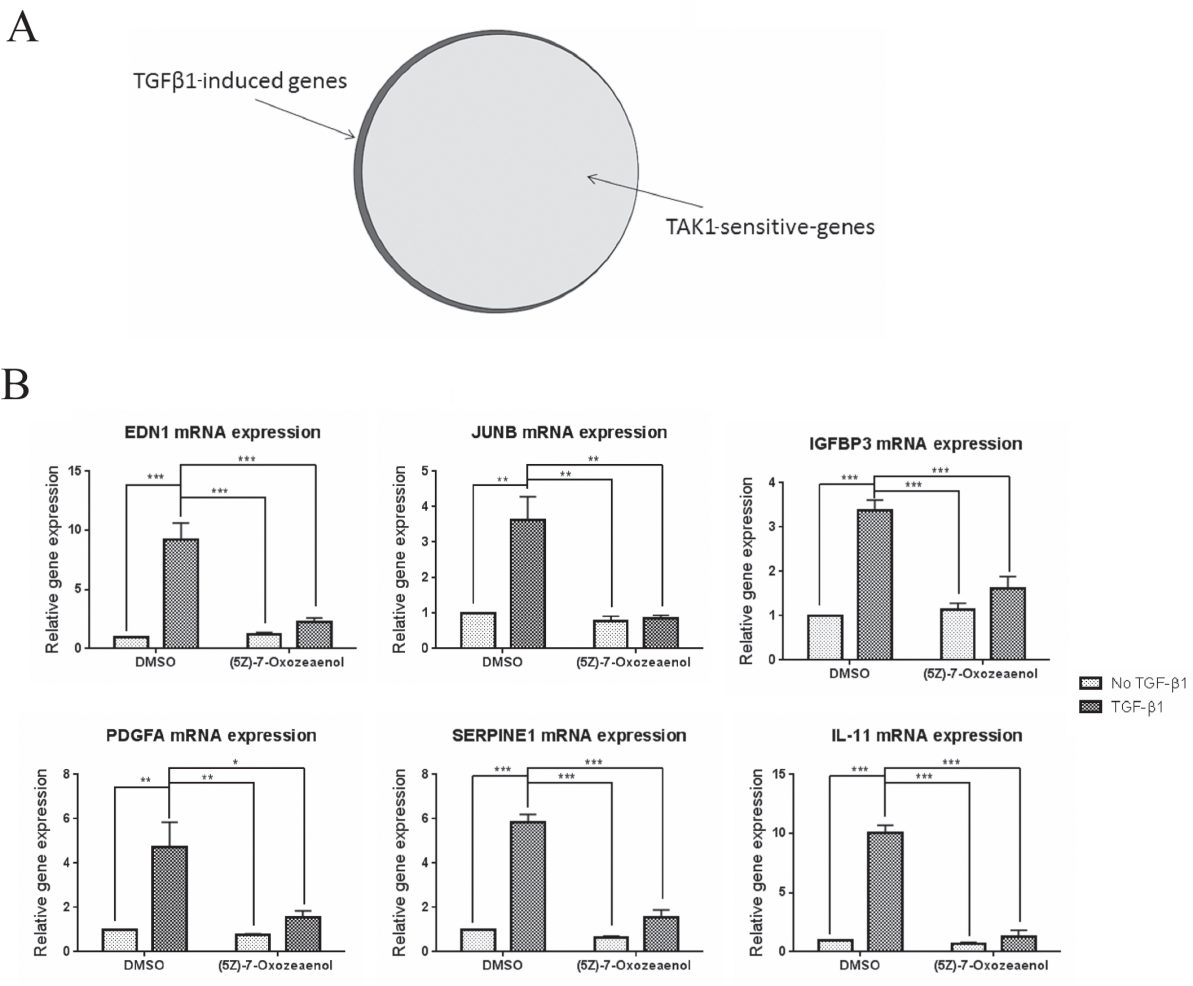
(5Z)-7-oxozeaenol inhibits TGFβ1-induced mRNA expression in human gingival fibroblasts. (A) Human gingival fibroblasts were serum starved overnight and pre-treated with (5Z)-7-oxozeaenol (400 nM) or DMSO for 45 min followed by treatment with TGFβ1 (4ngml^-1^ (90 pM)) ligand or left untreated. Total RNA was harvested six hours later and subjected to gene expression profiling using GeneChip Human Gene 1.0 ST arrays (N = 2) as described in Methods. 147 genes were up-regulated in response to TGFβ1 (1.7 fold induction compared to DMSO control group) and 139 genes of the latter group were found to be (5*Z*)-7-Oxozeaenol sensitive. (B) Human gingival fibroblasts were treated as in (A) and subject to TaqMan RT-qPCR analysis using the indicated probe/primer set. 18S RNA was used as the internal control. (N = 3; averages+/-SEM are shown. * = p<0.05; ** = p<0.01; *** = p<0.001, One-Way ANOVA).

To verify the extent to which TAK1 is required for the induction of TGFβ1-responsive mRNAs in human gingival fibroblasts, cells were treated with or without TGFβ1 (6 hours, 4 ng/ml) in the presence of either (5*Z*)-7-Oxozeaenol inhibitor or DMSO (vehicle). RNAs were extracted and subjected to gene expression profiling using GeneChip Human Gene 1.0 ST arrays. Of the 28,869 genes on the Human GeneChip Gene 1.0 ST Arrays, 147 genes were up-regulated greater or equal to 1.7-fold in response to TGFβ1. Of these, 139 genes were (5*Z*)-7-Oxozeaenol-sensitive, suggesting that TAK1 mediates essentially all transcriptional responses to TGFβ1 in gingival fibroblasts (Fig 4A). Functional cluster analysis of the 139 (5*Z*)-7-Oxozeaenol-sensitive mRNAs revealed that those genes affected were included in clusters involved in a hyperproliferative fibrotic response, including mRNAs encoding genes involved with migration/cell adhesion, wound healing and cell cycle/proliferation (Table 1).

**Table 1:** Cluster analysis of TAK1 depended mRNAs with over 1.7 fold induction (average of two arrays) in response to TGFβ-1 treatment.

cell cycle/proliferation cluster				
**Affymetrix ID**	**GENBANK ACCESSION**	**Gene name**		**Fold Increase**
**8116921**	AK291838	endothelin 1	EDN1	6.7818
**8024485**	AF078077	growth arrest and DNA-damage-inducible, beta	GADD45B	2.39958
**8139207**	AK290584	inhibin, beta A	INHBA	2.04306
**8026047**	BC004250	jun B proto-oncogene	JUNB	1.87672
**7922976**	AK292167	prostaglandin-endoperoxide synthase 2 (prostaglandin G/H synthase and cyclooxygenase)	PTGS2	1.92186
**8147012**	BC022265	protein kinase (cAMP-dependent, catalytic) inhibitor alpha	PKIA	2.41012
**8040473**	AF498971	ras homolog gene family, member B	RHOB	1.88318
**8010061**	AF200328	sphingosine kinase 1	SPHK1	1.72518
motion, migration, adhesion cluster				
**Affymetrix ID**	**GENBANK ACCESSION**	**Gene name**	** **	**Fold Increase**
**8023220**	AF010193	SMAD family member 7	SMAD7	2.841
**8160637 **	AK297541	UDP-Gal:betaGlcNAc beta 1,4- galactosyltransferase, polypeptide 1	B4GALT1	2.12002
**8116921**	AK291838	endothelin 1	EDN1	6.7818
**8072678**	BC001491	heme oxygenase (decycling) 1	HMOX1	2.05866
**8114572**	BC033097	heparin-binding EGF-like growth factor	HBEGF	4.0607
**8139488**	AK298143	insulin-like growth factor binding protein 3	IGFBP3	1.88748
**8137670**	AK292217	platelet-derived growth factor alpha polypeptide	PDGFA	2.07886
**8010061**	AF200328	sphingosine kinase 1	SPHK1	1.72518
**8148304**	AF205437	tribbles homolog 1 (Drosophila)	TRIB1	2.96456
**7962579**	AY454159	adhesion molecule with Ig-like domain 2	AMIGO2	3.45511
**8022674**	BC036470	cadherin 2, type 1, N-cadherin (neuronal)	CDH2	2.04821
**8035517**	AK074508	cartilage oligomeric matrix protein	COMP	3.02008
**8121685**	AJ420528	discoidin, CUB and LCCL domain containing 1	DCBLD1	1.85226
**8056184**	AK290300	integrin, beta 6	ITGB6	2.41065
**8102232**	AF198532	lymphoid enhancer-binding factor 1	LEF1	1.90975
**8123936**	AK292682	neural precursor cell expressed, developmentally down-regulated	NEDD9	2.93624
**8047738**	AL833606	neuropilin 2	NRP2	1.73487
**8040473**	AF498971	ras homolog gene family, member B	RHOB	1.88318
Wound healing cluster				
**Affymetrix ID**	**GENBANK ACCESSION**	**Gene name**		**Fold Increase**
**7950933**	AB041035	NADPH oxidase 4	NOX4	8.49418
**8160637**	AK297541	UDP-Gal:betaGlcNAc beta 1,4- galactosyltransferase, polypeptide 1	B4GALT1	2.12002
**8072678**	BC001491	heme oxygenase (decycling) 1	HMOX1	2.05866
**8114572**	BC033097	heparin-binding EGF-like growth factor	HBEGF	4.0607
**8056184**	AK290300	integrin, beta 6	ITGB6	2.41065
**8039484**	AK290572	interleukin 11	IL11	1.84543
**8137670**	AK292217	platelet-derived growth factor alpha polypeptide	PDGFA	2.07886
**8135069**	AK293248	serpin peptidase inhibitor, clade E (nexin, plasminogen activator inhibitor type 1), member 1	SERPINE1	2.17387

Genes involved with a hyperproliferative response are shown.

Of the genes detected by microarray analysis to be sensitive to TAK1 inhibition, junB transcription factor (JUNB), platelet-derived growth factor-α (PGDFA), endothelin-1 (EDN1), insulin-like growth factor binding protein 3 (IGFBP3), plasminogen activator-inhibitor-1 (PAI-1/SERPINE1), Platelet-derived growth factor-α-polypeptide (PDGFA) and interleukin-11 (IL-11) were selected for further analysis and validation by real time PCR. Endothelin-1 and junB were selected based on their known association in fibrosis and in pro-fibrotic programming in response to TGFβ [21–24]. PGDFA was selected as this protein promotes tissue repair and fibrosis [25]. Moreover, IGFBP-3 promotes TGFβ-dependent stromal remodeling [26], PAI-1/SERPINE1 contributes to excessive collagen deposition in wounds [27] and IL-11 is involved with TGFβ signaling and pulmonary myofibroblast activation [28]. In all cases, real time PCR analysis verified the microarray data showing that induction of these mRNAs in response to TGFβ was reduced by (5*Z*)-7-Oxozeaenol (Fig 4B). Conversely, baseline (i.e., uninduced) mRNA expression was not significantly affected by (5*Z*)-7-Oxozeaenol (Fig 3B).

### (5*Z*)-7-Oxozeaenol reduces gingival fibroblast proliferation

**Fig 5:**
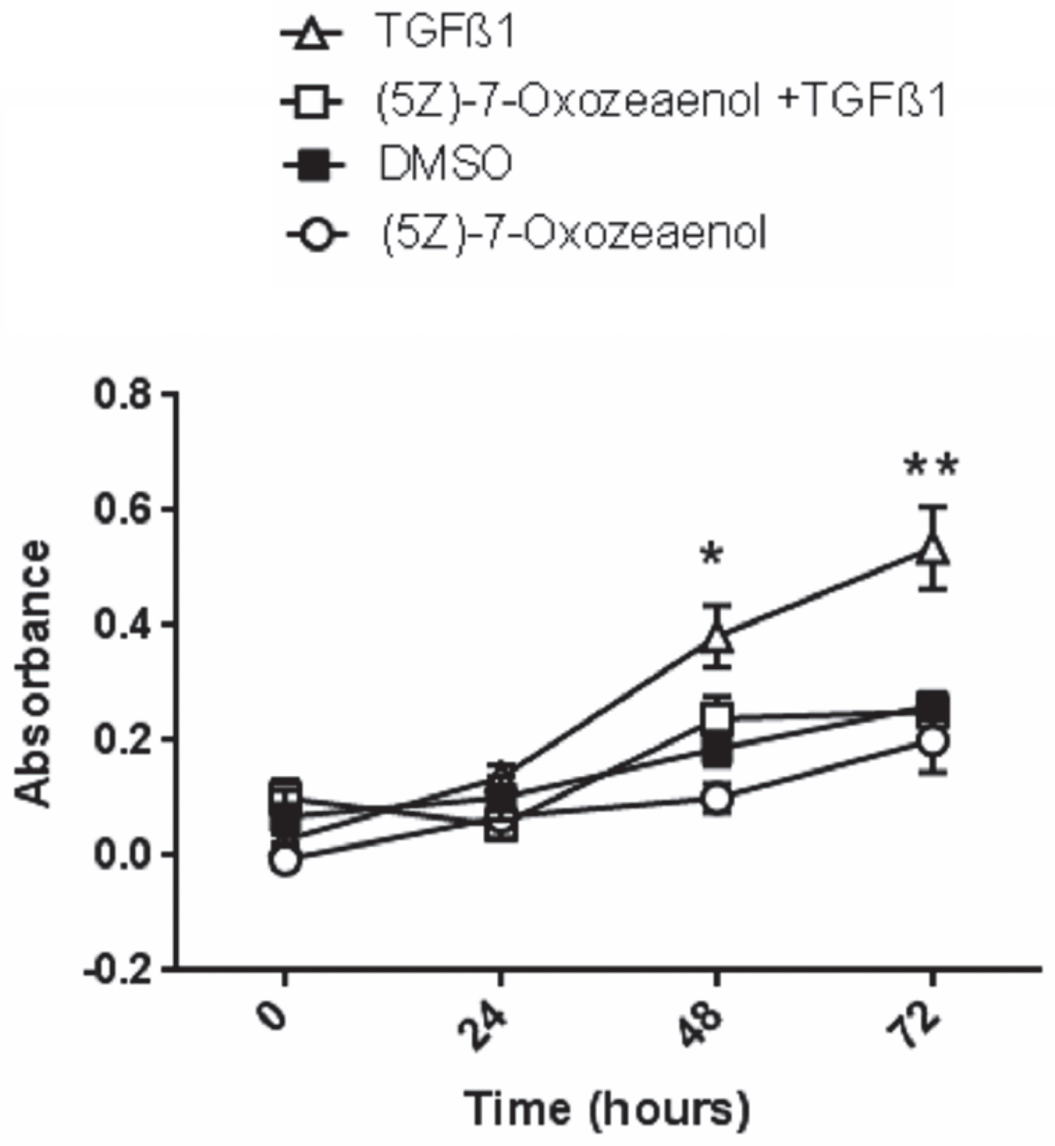
(5Z)-7-oxozeaenol reduces TGFβ1 induced gingival fibroblast proliferation. Human gingival fibroblasts were serum starved overnight and pre-treated with (5Z)-7-oxozeaenol ((5Z)-7-oxo; 400 nM) or DMSO for 45 min followed by treatment with TGFβ1 (4ngml-1 (90 pM)) ligand or left untreated. Cultures were grown in the presence of BrdU for up to 72 hours as described in methods. One of three representative experiments is shown; (N = 4; averages+/-SEM are shown * p<0.05 for: DMSO vs TGFβ1, (5Z)-7-oxo vs TGFβ1, TGFβ1 vs (5Z)-7-oxo+TGFβ1; ** p<0.05 for: DMSO vs TGFβ1; (5Z)-7-oxo vs TGFβ1, TGFβ1 vs (5Z)-7-oxo+TGFβ1. Two-Way ANOVA followed by Tukey's Post Hoc analysis).

Having established that the cluster involved with proliferation was affected by (5*Z*)-7-Oxozeaenol, we performed a functional validation of this result using a BrdU-based proliferation assay (Fig 5). Compared to DMSO alone, TGFβ1 treatment resulted in increased proliferation that was sensitive to (5*Z*)-7-Oxozeaenol. These results are consistent with the hypothesis that TAK1 controls fibroproliferative responses in gingival fibroblasts

## Conclusions

TGFβ promotes wound healing and fibrotic responses in vitro and in vivo [5]. Fibroblast activation in response to TGFβ1 involves both canonical ALK5/Smad-dependent and non-canonical Smad-independent pathways [5]. An example of a non-canonical pathway is the TAK1 pathway, which mediates p38 and JNK phosphorylation in response to TGFβ [29]. Previously, we used the selective TAK1 inhibitor 5Z-7-Oxozeanol to show that, in human dermal fibroblasts, the non-canonical TAK1 pathway mediates the induction of ~70% of the mRNAs in response to TGFβ1, including pro-fibrotic gene expression clusters [13]. Specifically, in this previous study, induction of 741/1049 TGFβ-inducible transcripts were sensitive to (5*Z*)-7-Oxozeaenol and wound healing and ECM clusters were found to be sensitive to 5Z-7-Oxozeanol [13]. In this report, we extend these prior reports by using 5Z-7-Oxozeanol to show that the non-canonical TAK1 pathway mediates essentially all transcriptional responses to TGFβ1 in gingival fibroblasts. 5Z-7-Oxozeanol also blocked the ability of TGFβ-induced CCN2 mRNA and protein expression as well TGFβ-induced collagen type I protein and proliferation. Thus the TAK1 pathway is operant in gingival fibroblasts and mediates fibroproliferative responses to TGFβ in this cell type.

Unlike skin, gingiva do not scar in response to fibrogenic stimuli. Fibrotic responses in gingival fibroblasts are largely fibroproliferative but not fibrocontractile and hence are neither characterized by scar tissue or by abundant myofibroblasts, the cell type believed to be responsible for scar tissue formation and chronic fibrosis [30–32]. It is interesting to note, however, that in both dermal and gingival fibroblasts, the TAK1-mediated pathway operates and, in both cell types, mediates fibroproliferative responses to TGFβ. Thus a failure of the TAK1 pathway to operate in gingival fibroblasts is not likely to be the basis underlying scarless repair. An emerging body of evidence links excess mechanical loading/stimulation to scar tissue formation [33–36]. Indeed, gingival fibroblasts inherently show reduced expression of pro-adhesive and pro-contractile genes both basally and in response to TGFβ; these features have been linked to reduced activation of the pro-adhesive signaling pathway [16, 30, 37]. Hence alterations in expression of mechanotransductive proteins may underlie basis for scarless repair. Nonetheless, our current results are consistent with the hypothesis that TAK1 inhibitors such as (5Z)-7-Oxozeanol may be considered, in the future, for treating disorders characterized by fibroproliferative responses such as gingival hyperplasia.
